# Editorial

**DOI:** 10.1007/s10493-012-9542-0

**Published:** 2012-03-15

**Authors:** Maurice W. Sabelis

**Affiliations:** IBED, Section Population Biology, University of Amsterdam, Amsterdam, The Netherlands

I accepted the task to act as Editor-in-Chief for *Experimental and Applied Acarology* (EAA), as per January 2012. I have used this opportunity to change and extend the Editorial Board and to broaden the scope of the journal by explicitly inviting papers in such fields as evolutionary biology, behavioural ecology, molecular biology and experimental evolution of mites and ticks, also focusing on all the ways in which they interact with other organisms (plants, arthropods and other animals). I realize I face a task with heavy responsibilities, even more so because the journal has built an excellent reputation. The number of submissions has increased with the years (e.g. 8% in 2010), the rejection rate has gone up (to 42% in 2010), the impact factor has risen (to 1.825 in 2010) and as a consequence the journal now ranks at position 16 out of all 83 journals in the field of Entomology. A salient feature of EAA is the space offered for special issues that provide reviews on various topics of particular relevance to Acarology. In my view the publication of special issues has to be stimulated, the rejection rate should increase and the time from submission to first decision should be reduced (66 days in 2010). Together with two Managing Editors, Dr Frans Jongejan for papers on ticks and Dr Jan Bruin for papers on mites, I am confident that we will be able to create an even stronger journal in years to come.

This editorial could not have been written at a more dramatic moment for EAA. On February 8, 2012 Professor Wim Helle, founding editor of EAA, is deceased. He had the vision and the initiative to start a journal in 1985 with a focus on experimental and applied biology of mites and ticks at a time where other acarological journals emphasized taxonomy and systematics. Dr Leo van der Geest has written an obituary. We will always remember Wim Helle as a scientist who made significant contributions to the genetics of mites (pesticide resistance, diapause, haplodiploidy, genetic systems and reproductive incompatibility). He not only created EAA, he also initiated the book series on World Crop Pests (with two volumes on spider mites and one on eriophyoid mites), and was among the first to establish the European Association of Acarologists (EURAAC).

